# Brain Mysteries: Complexity beyond Imagination

**DOI:** 10.1523/ENEURO.0429-23.2023

**Published:** 2023-11-14

**Authors:** Christophe Bernard

I am always marveled by the brain’s vast capabilities. Each discovery, each revelation, seems to open a door only to reveal myriad others yet unopened. The brain, with its vast intricacies, consistently reminds us that its complexity transcends the limits of our imagination. It is in this spirit that I wish to introduce “Brain Mysteries: Complexity Beyond Imagination” in *eNeuro*.

All neuroscientists have been contributing for decades to the mapping of brain networks and to the unraveling of its codes. While every stride has been fruitful in terms of knowledge, with each answer, newer, deeper questions have arisen. Just when we believe we are nearing an understanding, the brain unveils another layer of its profound intricacy.

In this series, we dare to venture into the depths of these layers. “Brain Mysteries: Complexity Beyond Imagination” is not just a sequence of articles: it is a call to think beyond, to challenge our existing knowledge, and to embrace the vastness of what we do not yet know. We will delve into questions that are seldom addressed in mainstream neuroscientific discourse, topics that are perplexing, intriguing, and, at times, unsettling.

The first papers will deal with degeneracy (Bernard, 2023), with a topical twist in relationship with global warming (Marder, 2023), a central biological issue that remains to be accounted for in our research strategies. At the end of the spectrum lies the question, what is consciousness or sentience, a question that I wish will open a debate (Rouleau and Levin, 2023; Gomez-Marin, 2023). But science is not only having ideas and hypotheses and testing them, it starts with the means to do it. Should big challenges be addressed with big money? Building on the outcome of the Human Brain Project, Yves Frégnac (Frégnac, 2023) will develop what could or should be done.

This initial set of papers will be followed by others, including the forthcoming “Opening the Pandora’s box of brain–body relationships” by Catherine Tallon-Baudry.

To you, fellow neuroscientists, we offer a challenge: engage with this series not as mere observers but as explorers and contributors to the series. At *eNeuro*, we believe that pushing boundaries and exploring the unknown is the essence of scientific endeavor. “Brain Mysteries” embodies this belief. As Marcel Proust wrote: “Le véritable voyage de découverte ne consiste pas à chercher de nouveaux paysages, mais à avoir de nouveaux yeux.” (“The real voyage of discovery consists not in seeking new landscapes, but in having new eyes.”).
